# Examining the potentials of stem cell therapy in reducing the burden of selected non-communicable diseases in Africa

**DOI:** 10.1186/s13287-024-03864-4

**Published:** 2024-08-13

**Authors:** Faith Ayobami Atewologun, Olalekan John Okesanya, Inibehe Ime Okon, Hassan Hakeem Kayode, Bonaventure Michael Ukoaka, Noah Olabode Olaleke, Jerico Bautista Ogaya, Lawal Azeez Okikiola, Emery Manirambona, Don Eliseo Lucero-Prisno III

**Affiliations:** 1 Department of Medicine and Surgery, Ladoke Akintola University of Technology, Ogbomoso, Nigeria; 2 Faculty of Laboratory Hygiene and Epidemiology, University of Thessaly, Volos, Greece; 3 Department of Research, Medical Research Circle (MedReC), Democratic Republic of the Congo, Postal Code 50 Goma, Bukavu, Democratic Republic of Congo; 4 Department of Medical Laboratory Science, Federal Medical Centre, Bida, Niger State Nigeria; 5 Department of Internal Medicine, Asokoro District Hospital, Abuja, Nigeria; 6 Obafemi Awolowo University Teaching Hospital Complex, Ile-Ife, Osun State Nigeria; 7 Department of Medical Technology, Far Eastern University, Manila, Philippines; 8 Center for University Research, University of Makati, Makati City, Philippines; 9 Department of Biology, University of Texas at Tyler, Tyler, USA; 10 Department of Medical Laboratory Science, Kwara State University, Malete, Nigeria; 11 College of Medicine and Health Sciences, University of Rwanda, Kigali, Rwanda; 12 Department of Global Health and Development, London School of Hygiene and Tropical Medicine, London, UK; 13 Research and Innovation Office, Southern Leyte State University, Sogod, Southern Leyte Philippines

**Keywords:** Stem cell therapy (SCT), Mesenchymal stem cells (MSCs), Neural stem cells (NSC), Hematopoietic stem cells (HSCs), Pluripotent stem cells (PSCs), Cell-based therapy, Regenerative medicine

## Abstract

Stem cell therapy (SCT) is a promising solution for addressing health challenges in Africa, particularly non-communicable diseases (NCDs). With their regenerative potential, stem cells have the inherent capacity to differentiate into numerous cell types for tissue repair. Despite infrastructural, ethical, and legal challenges, SCT holds immense promise for managing chronic illnesses and deep-seated tissue injuries. The rising prevalence of NCDs in Africa highlights the need for innovative strategies and treatment options. SCT offers hope in combating conditions like burns, osteoarthritis, diabetes, Alzheimer’s disease, stroke, heart failure and cancer, potentially reducing the burden of NCDs on the continent. Despite SCT’s opportunities in Africa, there are significant obstacles. However, published research on SCT in Africa is scarce, but recent initiatives such as the Basic School on Neural Stem Cells (NSC) express interest in developing NSC research in Africa. SCT research in African regions, notably on neurogenesis, demonstrates a concentration on studying neurological processes in indigenous settings. While progress has been made in South Africa and Nigeria, issues such as brain drain and impediments to innovation remain. Clinical trials have investigated the efficacy of stem cell treatments, emphasising both potential benefits and limitations in implementing these therapies efficiently. Financing research, developing regulatory frameworks, and resolving affordability concerns are critical steps toward realizing the potential of stem cell treatment in Africa.

## Introduction

Stem cells, basic undifferentiated cells, hold great potential in regenerative medicine. There are promises for Africa’s tissue regeneration and engineering, which could be revolutionised by stem cell therapy (SCT) [1, 2]. SCT stimulates, regulates, and modulates the body’s endogenous stem cells while rejuvenating tissues and maintaining their original characteristics [1]. They are an essential component of medical research and therapy and are categorised based on their origin as embryonic stem cells (ESCs), induced pluripotent stem cells (iPSCs), or mesenchymal stem cells (MSCs) [2]. By harnessing the clinical potential of stem cells and their derivatives, SCT stimulates the body’s innate healing capabilities to repair damaged and malfunctioning tissues [3]. They replace damaged cells and tissues, perhaps eliminating the need for costly, lifelong treatment modalities [3, 4]. Africa’s health issues could be resolved by leveraging stem cells’ diagnostic and therapeutic capabilities to cure various chronic illnesses, wounds, and impairments [3, 5]. Stem cells can differentiate into cell types to repair damaged tissues, such as neurons, cardiac, and liver cells, based on the body’s needs [4, 5]. They are classified as totipotent, multipotent, unipotent, or pluripotent based on their differentiating capacity [2, 3, 5]. Additionally, they have anti-inflammatory qualities, promote epithelial cell growth, and stop wounds from scarring, while the neurological system and bone tissue regeneration are significantly aided [5]. However, despite its enormous potential advantages, infrastructural, ethical, and legal considerations must be addressed to guarantee its safe and efficient clinical application [4].

**Fig. 1:**
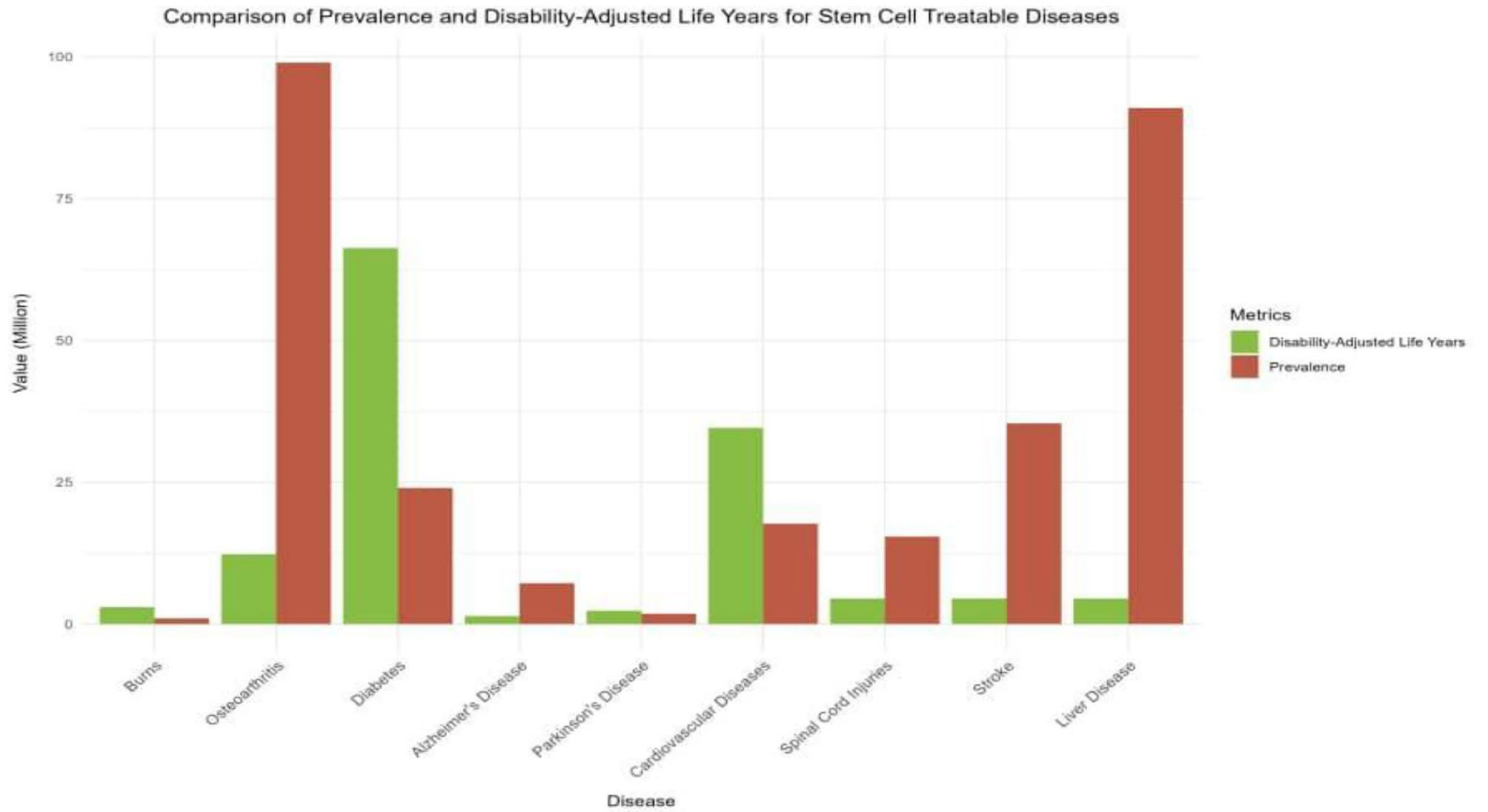
Comparison of prevalence and disability-adjusted life years for stem cell treatable diseases. [6–12]

**Fig. 2:**
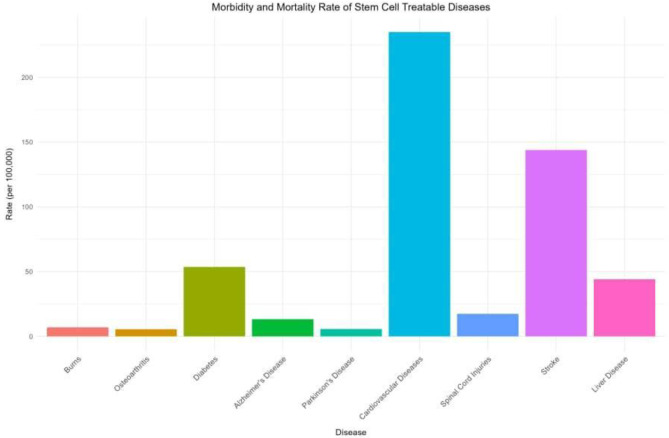
Morbidity and mortality rate of stem cell treatable diseases [6–12, 16]

The burden of non-communicable diseases (NCDs) is becoming a global and public health concern, especially in Africa. Conditions such as burns, osteoarthritis, cancer, cardiovascular diseases (CVDs), diabetes, Alzheimer’s disease, spinal cord injuries, heart failure and stroke are increasing, posing a substantial threat in the region (Fig. 1) [13]. These diseases are becoming more prevalent than communicable diseases as the leading cause of death among those under the age of 70, fuelled by recent socioeconomic, demographic, and epidemiological dynamics in the region (Fig. 2) [13, 14]. The World Health Organisation (WHO) estimates that NCDs cause an annual loss of nearly 2.4 trillion dollars to Africa’s gross domestic product. [6, 7]. Consequently, to address this trend, research on SCT potentials is recommended [14, 15]. Significant advancements have been made in developing technologies to tackle diseases in Africa, even though most gene therapy research is being carried out outside the continent. This limits the long-term feasibility of addressing NCDs in Africa, including the ability to treat inherited diseases, eradicate cancer, and inactivate viruses. A comprehensive analysis is crucial to assess the effectiveness of SCT and identify opportunities to enhance region-specific medical interventions [15]. Thus, this study aims to explore the current state, challenges, and potential clinical applications of stem cell therapy for NCDs in Africa.

### Historical development and advancement of stem cell therapy

Exploring the field of stem cell biology, Ernst Haeckel, a well-known German biologist, used the phrase “stem cell” in 1868 to delineate the prodigious capacity of fertilized eggs to produce all cells in an organism [17]. This discovery marked the beginning of the field of stem cell biology. In 1888, Theodor Heinrich Boveri and Valentin Haecker, who were German zoologists, described stem cells as a distinct cell population within embryos capable of developing into specialised cells, marking the first inception of stem cell treatment [18]. In 1902, histologist Franz Ernst Christian Neumann and chemist Alexander Alexandrowitsch Maximov discovered common progenitor cells and developed the concept of polyblasts, which were later known as stem cells by Haeckel [19, 20]. Maximov’s research highlighted the bone marrow’s hematopoietic potential [20]. A crucial preliminary advancement in using stem cell treatment in medicine was the 1939 case report detailing the human bone marrow transplant to cure an aplastic anaemia patient [21].

In 1958, French oncologist George Mathe performed the first stem cell transplant, a significant milestone in the history of the process. This procedure involved using bone marrow transplantation for the treatment of six nuclear researchers who had suffered from harmful radiation exposure at work [22]. Furthering the scientific understanding of SCT, Mathe’s groundbreaking work was continued in 1963 when he successfully performed a bone marrow transplant on a patient diagnosed with leukaemia [23]. Meanwhile, the pioneering method of allogeneic hematopoietic stem cell transplantation (HSCT) was undertaken by Dr. E. Donnall Thomas, who performed the first successful allogeneic transplant in 1957. Nevertheless, the early attempts were not without difficulties. The ambiguities and risks surrounding bone marrow transplantation led to high mortality rates and poor engraftment success in the earliest investigations [24]. Despite these obstacles, considerable advancements were made; for example, the success of allogeneic transplantation for diseases like acute myeloid leukaemia and aplastic anaemia was made possible by the discovery of cyclosporine in 1972 [25]. The first umbilical cord blood stem cell transplant was subsequently carried out in 1988 on a kid suffering from Fanconi’s anaemia, signalling the start of a new era in stem cell transplantation. A major step towards the general availability of SCT was the formation of the first public and private stem cell banks in the United States in 1992 [26, 27]. Later research revealed that these cells differed from the hematopoietic population and proliferated quickly in tissue culture vessels as adherent cells. The research team observed the ability of these cells to differentiate into osteoblasts, adipocytes, and chondrocytes in suspension culture, following their capability to generate colony-forming units (CFUs) from bone marrow. The term “mesenchymal stem cells” subsequently replaced earlier terms like “osteogenic” or “stromal stem cells” after the discovery of human embryonic stem cells (hESCs) in 1991 [28].

The past decades have witnessed notable advances in SCT research and development. Evans, Kaufman, Smithies, and Capecchi were awarded the 2007 Nobel Prize in Physiology or Medicine for their pioneering work in stem cell biology, which included the successful production of mouse embryonic stem cells in a lab setting in 1981 [27]. The discovery of human induced pluripotent stem cells (iPS) in 2007 and human embryonic stem cells by James Alexander Thomson in 1998 expanded the scope of stem cell therapies and research. The field’s progress has led to significant advancements, such as the therapeutic potential of umbilical cord stem cells, which are now seen as equally valuable as bone marrow stem cells [29]. The first report on adult stem cell transplantation from umbilical cords was published in 2001, sparking further research into their clinical applications. In 2004, Gesine Koegler and associates discovered pluripotent stem cells in umbilical cord blood, adding to the growing body of evidence supporting the therapeutic potential of stem cells [30]. Shinya Yamanaka and John Gurdon’s 2012 Nobel Prize in Physiology or Medicine recognised their groundbreaking discovery that mature cells could be reprogrammed into stem cells, opening new possibilities for stem cell manipulation and pluripotency [31].

Advancements in stem cells and regenerative medicine have revolutionised the biomedical sector since the 20th century, fostering the management of NCDs, and extending human life expectancy and overall quality of life (Tables 1 and 2) [32]. Blastocyst-derived adult stem cells (ASCs) and embryonic stem cells (ESCs) have emerged with exceptional therapeutic features. In animal investigations, ESCs have fostered brain regeneration, indicating they can self-regenerate and differentiate into all tissue types in vivo [4]. Derived from the blastocysts’ inner cell mass (ICM), pluripotent human embryonic stem cells (hESCs) can differentiate into any cell type in the body. Thereafter, they become multipotent stem cells that differentiate into numerous cell types found in particular germ layers [3]. In contrast, adult stem cells with the capacity for multidirectional differentiation include MSCs obtained from the early mesoderm [33]. These are microscopically extracted from bone marrow, dental pulp, adipose tissue, umbilical cord, and other tissues. The advantages of MSCs include their low immunogenicity, high expansion potential, and ease of isolation. In addition to hESCs and MSCs, other stem cell types, such as urine-derived stem cells, neural stem cells (NSCs), dental stem cells (DSCs), and myogenic stem cells (MDSCs), offer promising pathways for tissue regeneration [34].

**Table 1:** List of completed clinical trials on the use of Stem Cell Therapy in non-communicable diseases

NCT NUMBERS	References	Study Title	Study Status	Brief Summary	Interventions	Sponsor	Study Type
		**Arthritis and Musculoskeletal system studies/interventions**					
NCT03308006	[35]	Knee Osteoarthritis Treatment with Adipose-derived Stem Cells: Phase II Clinical Trial	Completed	The existing management of OA is conservative and pharmacological, aiming to relieve symptoms of osteoarthritis and improve joint function. Stem cells will be separated from fat cells in the adipose tissue and then activated in the Tissue Culture Lab	Biological: Stem cells	King Faisal Specialist Hospital & Research Centre, Jeddah	Interventional
NCT03264573	[36]	Role of Stem Cells, Platelet-Rich Plasma in Treatment of Scars	Completed	The objective of this study is to establish a protocol for post-scar revision care and to study the effect of PRP and/or adipose-derived mesenchymal stem cell injection on the improvement of atrophic scars after scar revision.	Biological: Stem cell Device: MSCs	Heba Mohamed Saad Eldien	Interventional
		**Gastro-intestinal studies/interventions**					
NCT05210309	[37]	National Project to Implement Mesenchymal Stem Cell for the Treatment of Perianal Crohn’s Fistula (the PRIME Study)	Completed	Darvadstrocel is an expanded allogeneic adipose-derived mesenchymal stem cell therapy for the treatment of complex perianal fistulas in patients with Crohn’s disease. The objective is to get a homogeneous implementation in all hospitals in Spain that have been baked to use this biological therapy.	Procedure: Stem Cell Therapy	Universidad de Zaragoza	Observational
NCT03466515	[37]	Stem Cells Treatment of Complex Crohn’s Anal Fistula	Completed	To investigate the safety and feasibility of stem cell treatment of complex anal fistula in patients with Crohn’s disease.	Procedure: stem cell injection	University of Southern Denmark	Interventional
		**Central nervous systems studies/interventions**					
NCT04484402	[38]	Treatment of Patients With Inflammatory-Dystrophic Diseases of the Cornea Using Autologous Stem Cells	Completed	This study aims to ensure the treatment of patients with inflammatory-dystrophic diseases of the cornea using autologous limbal stem cells (corneal epithelial stem cells) or adipose-derived mesenchymal stem cells	Biological: Mesenchymal stem cells Biological: limbal stem cells Others: standard treatment	Institute of Biophysics and Cell Engineering of National Academy of Sciences of Belarus	Interventional
		**Pulmonary studies/interventions**					
NCT02443961	[39]	Mesenchymal Stem Cell Therapy for Bronchopulmonary Dysplasia in Preterm Babies	Completed	MSC therapy in patients at high risk of BPD prevents pulmonary lesions.	Biological: Mesenchymal Stem Cell (MSC) therapy	Fundacion para la Investigacion Biomedica del Hospital Universitario Ramon y Cajal	Interventional
NCT04713878	[40]	Mesenchymal Stem Cells Therapy in Patients With COVID-19 Pneumonia	Completed	To Provide immune modulation by Stem Cell Transplantation and reducing the damage caused by cytokine storms to tissues and organs.	Others: Mesenchymal stem cells	Kanuni Sultan Suleyman Training and Research Hospital	Interventional
NCT04062136	[40]	Umbilical Cord Mesenchymal Stem Cells Transplantation in the Treatment of Bronchopulmonary Dysplasia	Completed	To evaluate the safety and efficacy of human umbilical cord mesenchymal stem cell transplantation in patients with bronchopulmonary dysplasia	Combination product: stem cell transplantation	Vinmec Research Institute of Stem Cell and Gene Technology	Interventional
NCT03044431	[41]	Autologous Stem Cell Treatment for Chronic Lung Disease Study	Completed	The purpose of the Lung Institute is to collect and isolate a patient’s cells and platelet-rich plasma (PRP) and deliver the product back to the patient the same day.	Procedure: Cell therapy	Lung Institute	Observational
		**Others**					
NCT02824393	[42]	Experimental Autologous Mesenchymal Stem Cell Therapy in Treatment of Chronic Autoimmune Urticaria	Completed	This study aims to determine whether autologous adipose tissue-derived mesenchymal stem cells for the treatment of chronic autoimmune urticaria are safe and effective.	Biological: Autologous mesenchymal stem cell	Celal Bayar University	Interventional

**Table 2:** List of active clinical trials on the use of stem cell therapy in non-communicable diseases

NCT Number	Study Title	Study Status	Interventions	Sponsor	Phases	Study Type	Start Date
NCT03333486	Fludarabine Phosphate, Cyclophosphamide, Total Body Irradiation, and Donor Stem Cell Transplant in Treating Patients With Blood Cancer	Active	Drug: Cyclophosphamide Drug: Fludarabine Phosphate Other: Laboratory Biomarker Analysis Procedure: Peripheral Blood Stem Cell Transplantation Radiation: Total-Body Irradiation	Roswell Park Cancer Institute	Phase 2	Interventional	12/7/2017
NCT02797470	Gene Therapy in Treating Patients With Human Immunodeficiency Virus-Related Lymphoma Receiving Stem Cell Transplant	Active	Procedure: Autologous Hematopoietic Stem Cell Transplantation Drug: Carmustine Drug: Cytarabine Drug: Etoposide Other: Laboratory Biomarker Analysis Biological: Lentivirus Vector CCR5 shRNA/TRIM5alpha/TAR Decoy-transduced Autologous CD34-positive Hematopoietic Progenitor Cells Drug: Melphalan Procedure: Peripheral Blood Stem Cell Transplantation	AIDS Malignancy Consortium	Phase 1 & 2	Interventional	6/23/2016
NCT02452697	Ph2 NK Cell Enriched DCIs w/wo RLR9 Agonist, DUK-CPG-001 From Donors Following Allogeneic SCT	Active	Biological: NK Cell enriched-DLI only Biological: NK-DLI + DUK-CPG-001	Cristina Gasparetto	Phase 2	Interventional	6/8/2016
NCT02192359	Carboxylesterase-Expressing Allogeneic Neural Stem Cells and Irinotecan Hydrochloride in Treating Patients With Recurrent High-Grade Gliomas	Active	Biological: Carboxylesterase-expressing Allogeneic Neural Stem Cells Drug: Irinotecan Drug: Irinotecan Hydrochloride Other: Laboratory Biomarker Analysis Other: Pharmacological Study	City of Hope Medical Center	Phase 1	Interventional	3/7/2016
NCT03391466	Study of Effectiveness of Axicabtagene Ciloleucel Compared to Standard of Care Therapy in Patients With Relapsed/Refractory Diffuse Large B Cell Lymphoma	Active	Biological: Axicabtagene Ciloleucel Drug: Platinum-containing salvage chemotherapy (e.g., R-ICE) followed by high-dose therapy (e.g., BEAM) and autologous stem cell transplant in responders Drug: Cyclophosphamide Drug: Fludarabine	Kite, A Gilead Company	Phase 3	Interventional	1/25/2018
NCT03030261	Elotuzumab, Pomalidomide, & Dexamethasone (Elo-Pom-Dex) With Second Autologous Stem Cell Transplantation for Relapsed Multiple Myeloma	Active	Drug: Elotuzumab Drug: Pomalidomide Drug: Dexamethasone	Washington University School of Medicine	Phase 2	Interventional	22/11/2017
NCT04430894	KRDI in Transplant-Eligible MM	Active	Drug: Carfilzomib Drug: Isatuximab Drug: Lenalidomide Drug: Dexamethasone	Massachusetts General Hospital	Phase 2	Interventional	7/10/2020
NCT03246529	Phase III, Safety, Tolerability and Efficacy of Combination Treatment of BL-8040 and G-GSF as Compared to Placebo and G-CSF for the Mobilization of Hematopoietic Stem Cells for Autologous Transplantation in Subjects With MM	Active	Drug: BL-8040 1.25 mg/kg + G-CSF Drug: Placebo + G-CSF	BioLineRx, Ltd.	Phase 3	Interventional	3/23/2018
NCT02759731	Study of Baricitinib, a JAK1/2 Inhibitor, in Chronic Graft-Versus-Host Disease After Allogeneic Hematopoietic Stem Cell Transplantation	Active	Drug: Baricitinib	National Cancer Institute (NCI)	Phase 1 & 2	Interventional	11/1/2016
NCT03786783	Dinutuximab, Sargramostim, and Combination Chemotherapy in Treating Patients With Newly Diagnosed High-Risk Neuroblastoma	Active	Procedure: Autologous Hematopoietic Stem Cell Transplantation Drug: Carboplatin Drug: Cisplatin Drug: Cyclophosphamide Drug: Dexrazoxane Biological: Dinutuximab Drug: Doxorubicin Drug: Etoposide Radiation: External Beam Radiation Therapy Drug: Isotretinoin Drug: Melphalan Biological: Sargramostim Drug: Thiotepa Drug: Topotecan Drug: Vincristine	National Cancer Institute (NCI)	Phase 2	Interventional	3/4/2019

### The current state of stem cell research in Africa

Research has been limited on NSCs in the African SCT research community. However, the inaugural Basic School on NSC, funded by the International Brain Research Organisation African Regional Committee IBRO-ARC, brought together early-career neuroscientists and clinicians from South Africa, Uganda, and Nigeria [15, 43], launching a new era in which scientists have explored the potential of SCTs for managing neurodegenerative illnesses and CNS regeneration. This has resulted in a growing body of research and a noticeable concentration of investigations in recent years. Currently, research is focused on neurogenesis using animal models, and laboratories in South Africa and Nigeria have actively investigated neurogenesis in rare African animals. Additionally, new research has begun to investigate the role of NSCs in human diseases, particularly rare familial syndromes, using translational and basic experimental methods [43, 44]. hESCs in innovative medication discovery and screening are changing the narrative in Africa, while ASCs are being experimented with bone marrow transplants for disorders such as myeloma [44, 45].

South Africa leads other countries in stem cell research, attracting patients from all over the continent seeking therapy for blood and cancer-related illnesses. Institutions like Netcare Femina Hospital in Pretoria, the University of Pretoria, and the University of Cape Town actively participate in stem cell research, demonstrating the country’s leadership in the field [45]. Egypt had a haematopoietic stem cell transplantation programme in 1989, a significant milestone in the region’s stem cell therapy landscape. This programme has evolved, with over 1320 transplants completed in the first 18 years of its operation [46, 47]. In Nigeria, the first successful stem cell transplant was performed in 2011 on a sickle cell anaemia patient at the University of Benin Teaching Hospital [48]. Since then, six more successful transplants have been reported. The Bone Marrow Registry, Nigeria (BMRN) was established in 2012 to improve global recovery statistics for Africans with blood disorders. BMRN plans to launch Africa’s first umbilical cord blood bank to support HSCT [49]. Stem cell research has also advanced in other African countries, such as Tunisia, Egypt, Morocco, Algeria, and Libya [49].

Current research and collaborations promise additional advances in stem cell therapy in Africa in the coming years. SCT in Africa reveals a substantial gap compared to other continents, with only a few clinical trials in the region. Currently, just 2.5% of the global mesenchymal stem cell clinical trials are conducted in Africa, starkly contrasting the statistics across North America, Asia and Europe [50]. Pluripotent stem cell clinical studies are also limited in Africa, with very few trials reported on the continent [50]. However, there is a rising interest in investigating the potential of stem cells in treating heart disorders [51]. Despite this, stem cells are mostly used as experimental models in vitro rather than for therapy, highlighting a need for additional research and development efforts to bridge the gap and fully realise the potential of stem cell treatment in Africa [51].

### Challenges to stem cell therapy in Africa

Numerous factors have challenged the use of SCT for treatment in Africa. Stem cell therapy in Africa faces challenges due to human capital flight and barriers to innovation [52]. Most countries in the region face a brain drain of skilled professionals, exacerbating healthcare inequities and hindering the establishment of robust cell therapy programs [53]. The inability to retain expertise also hinders the development of novel technologies. The innovation chasm between academic research and market-ready products remains a barrier to progress [53, 54]. Furthermore, financing for research in Africa frequently favours topics that are not as pertinent to the continent, which impedes the development of stem cell therapies [43]. The roadblocks to establishing SCT in Africa are diverse and go far beyond the formidable barrier posed by the high cost of treatment. While the expensive costs of licenced gene treatments make universal distribution nearly impossible in low and middle-income countries, additional barriers impede access to modern medical techniques [55]. For example, Glybera, being the first gene therapy approved in Europe, cost a startling €1 million and was finally withdrawn due to a lack of coverage in any jurisdiction, owing mostly to its prohibitively high cost [56]. Similarly, cancer immunotherapy, typified by the chimeric antigen receptor T cell (CAR-T) product, was initially priced at $475,000, and Zolgensma for spinal muscular atrophy became the most expensive medicine ever marketed, costing $2.125 million [55]. Beyond budgetary constraints, LMICs face significant gaps in healthcare infrastructure, such as inadequate neonatal and cancer screening programmes, poor electronic medical record systems, and limited laboratory diagnoses [57]. Furthermore, discrepancies in the distribution of standard-of-care treatments, together with common socio-cultural characteristics such as a preference for traditional healing approaches and a lack of medical literacy, limit timely diagnosis and intervention [55, 58].

Many countries are without national health insurance programmes, with patients relying on out-of-pocket payments or external funding for their healthcare needs [59]. Furthermore, basic care costs for NCDs already burden families’ resources, making the prospect of financing gene therapy much more difficult. The lack of affordability causes discrepancies in access, limiting treatment alternatives to the rich [60]. While some contend that gene therapy will eventually be more cost-effective than traditional disease management, current estimates indicate otherwise, highlighting the urgent need to investigate cost-cutting strategies. The Global Gene Therapy Initiative has proposed place-of-care bio-manufacturing as a potential option, subject to cooperation from global organisations such as the African Union and the WHO [61]. Another challenge is the absence of national guidelines for human gene therapy trials across many African countries. This deficiency poses obstacles to ensuring proper ethical and regulatory processes, particularly in South Africa, where existing regulatory mechanisms are reportedly contradictory and ambiguous [55]. Compounding this issue, the involvement of multiple national departments further complicates obtaining necessary ethics and regulatory approvals [62, 63].

### Potential application of stem cell therapy in Africa

#### Cardiovascular diseases (CVDs)

The potential use of SCT to treat CVDs has been thoroughly reviewed, with emphasis on hPSCs and MSCs [48]. Even in phase III trials, there is less evidence to demonstrate the effectiveness of stem cell therapies in increasing newborn size, heart function, or clinical outcomes, despite preclinical and clinical research having largely confirmed the safety of MSCs [31, 64]. A meta-analysis of 22 trials conducted by Hou et al., (2020), found that bone marrow-derived mononuclear cells significantly increased left ventricular ejection fraction and reduced infarct size in patients with acute myocardial infarction [65]. However, no significant cardiac function effects were observed based on myocardial contractility, cardiac remodelling, MRI-derived parameters or clinical outcomes from various stem cell sources [64, 65]. The unsatisfactory results may be associated with an intensified focus on immune regulation rather than regeneration. To determine the efficacy of these treatments, well-designed phase III studies with rigorous procedures, such as proper cell preparation, patient selection, re-evaluation protocols, and clinical assessments, are required [66].

### Gastrointestinal system disorders

Digestive tract diseases account for a considerable proportion of diagnoses in industrialised countries, affecting the lives of roughly one-third of people in the Western world [67]. The gastrointestinal system is protected by a single layer of epithelial cells, known for their strong regenerative capacity in response to injury and periodic cell turnover [67, 68]. These cells are capable of self-renewal and even faster with tissue damage and inflammation due to the presence of stem cells compartmentalised within intestinal crypts [67]. Exposure of intestinal stem cells to the gastrointestinal environment can cause direct depletion of the stem cell layer or disturbance of intestinal function, resulting in visible clinical signs [68].

Crohn’s disease and ulcerative colitis are the two most common inflammatory bowel diseases (IBD) [69]. Crohn’s disease is characterized by chronic, uncontrollable inflammation of the intestinal mucosa, with symptoms including segmental transmural mucosal inflammation, skip lesions and granulomatosis. Ulcerative colitis, on the other hand, is a chronic inflammatory condition that primarily affects the colon and rectum, with infection starting in the rectum and spreading up to the colon [70]. There are two main types of cellular therapy for Crohn’s disease: HSC-based therapy and MSC-based therapy [70]. Early case studies showed that HSC therapy could lead to long-term remission in some patients, which prompted further investigation. However, a large-scale randomized clinical trial (NCT00297193) conducted by Lindsay et al. (2017) between 2007 and 2011 found no significant differences in clinical outcomes compared to traditional therapy, and concerns about toxicity have been raised [71]. Despite the setbacks, systematic reviews and reevaluations of trial outcomes have shown some advantages of HSC therapy over controls in terms of disease activity index improvement [72]. HSCs are preferred over MSCs because they produce more consistent results, especially when obtained autologously [73].

### Liver disorders

Disruptions in liver homeostasis and function can result in a variety of chronic disorders, including liver failure, cirrhosis, cancer, alcoholic liver disease, nonalcoholic fatty liver disease (NAFLD), and autoimmune liver disease (ALD) [74]. While orthotopic liver transplantation remains the only viable treatment for severe liver disorders, the number of acceptable donor organs is extremely restricted. Currently, stem cell therapies for liver illness use HSCs, MSCs, hPSCs, and liver progenitor cells [74, 75]. Meta-analyses of clinical trials investigating SCT for acute-on-chronic liver failure (ACLF) have revealed short-term clinical advantages, with numerous doses of stem cells often required to extend therapeutic results [74, 76]. While MSC-based therapy has improved liver functions as measured by the end-stage liver disease score, albumin levels, total bilirubin, and coagulation, there has been no meaningful effect on survival rate or aminotransferase levels [76]. However, a randomised controlled trial by Lin et al. (2017) found that hepatic failure from viral hepatitis B treated with allogeneic bone marrow-derived MSCs (BM-MSCs) by peripheral infusion had improved liver function and fewer severe infections and substantially improved the 24-week survival rate in patients with HBV-related ACLF [75–77]. Liver cirrhosis has been managed with MSCs, and promising clinical outcomes have been reported [77, 78]. In a clinical trial, patients with chronic hepatitis B and decompensated liver cirrhosis were divided into two groups and given either umbilical cord-derived MSCs or a control treatment [78, 79]. The MSC group showed a significant reduction in ascites volume and improved liver function, as evidenced by higher serum albumin levels, lower total serum bilirubin levels, and decreased sodium model for end-stage liver disease score [79]. Similar results were seen in a phase II trial with bone marrow-derived MSCs among patients with HCV-induced liver cirrhosis [76, 78], as well as in three other clinical trials targeting liver cirrhosis caused by hepatitis B and alcoholic cirrhosis [79, 80]. Recent trials using bone marrow mononuclear cells (BMNCs) in children with liver cirrhosis post-Kasai surgery and in decompensated liver cirrhosis patients demonstrated safety and efficacy in improving liver function [80]. However, not all stem cell types or delivery strategies have been effective, as evidenced by a multinational phase 2 experiment in which CD133 + hematopoietic stem cell infusion failed to improve liver abnormalities [81].

Although preclinical studies have shown the efficacy of SCT for improved liver function in NAFLD models, human clinical trials have been limited [82]. A recent multicenter clinical trial (UMIN000022601) by Sakai et al. (2021) in Japan treated seven NAFLD patients with freshly extracted autologous adipose-derived stem cells. The data demonstrated elevated serum albumin levels in six individuals and increased prothrombin activity in five patients, with no treatment-related adverse events identified [83]. Currently, hematopoietic stem cell transplantation (HSCT) and bone marrow transplantation are the most common stem cell-based therapies that show therapeutic promise in autoimmune liver disease (ALD) clinical trials [57]. A study reported that haploidentical HSCTs effectively treated ALD in sickle cell patients, suggesting the potential of this method for dual disease treatment [53]. Clinical studies demonstrated that post-transplant immunosuppressive medication led to ALD remission. Primary biliary cholangitis (PBC), a subtype of ALD with progressive loss of bile ducts, has been treated with allogeneic umbilical cord-derived mesenchymal stem cells (UC-MSCs) and UDCA, with promising results [84]. In a Chinese trial involving ten PBC patients who had not responded to UDCA for over a year, allogeneic BM-MSCs showed good results with an intravenous infusion [69].

### Arthritis and burn treatment

Arthritis refers to a variety of cartilage-related disorders that cause joint discomfort and inflammation. The most common form is osteoarthritis (OA), for which SCT has recently emerged as a potential alternative treatment for osteoarthritis, attracting significant attention in regenerative medicine [85, 86]. HSCs have effectively reduced bone lesions, promoted bone regeneration, and stimulated rapid vascularization in degenerative cartilage [86]. Another study evaluating the efficiency of peripheral blood stem cells in ten OA patients by three intra-articular injections found a significant reduction in the WOMAC index, showing improvement across all criteria [87]. To improve the therapeutic potential of HSCT and CD34 + stem cells, they were recommended to be paired with a rehabilitation algorithm across preoperative, hospitalisation, and outpatient periods [87, 88]. Due to its immunoregulatory and anti-inflammatory qualities, MSC-based therapy for OA is currently gaining traction [88]. MSCs have been used as the principal cell source in multiple studies, revealing a favourable safety profile and potential efficacy in pain alleviation, cartilage degradation reduction, and cartilage structure and morphology regeneration in some cases [88, 89]. However, it is unclear whether bone marrow stem cells, adipose tissue, or the umbilical cord are the best sources of MSCs for OA therapy [89].

A comprehensive evaluation of 61 studies involving over 2390 OA patients found that MSC-based therapy had positive results, albeit with huge evidence and long-term monitoring. Another systematic analysis of 36 clinical trials, including 14 randomised trials, shed light on the potential of autologous MSC-based therapy for OA. Among trials using BM-MSCs, 57% reported significantly superior clinical results than the control group at the 1-year follow-up. Similar results were found for autologous adipose tissue-derived MSCs (AT-MSCs), indicating that clinical outcomes and MRI analysis provide insufficient evidence for MSC therapy’s therapeutic potential [88, 89]. The heterogeneity in outcomes could be attributed to differences in interventions, combination treatments, control treatments, and validated clinical outcome assessments among randomised clinical studies [90].

SCT has effectively treated burns by promoting tissue regeneration and reducing inflammation, promoting angiogenesis, collagen deposition, and cell proliferation, which all help with tissue repair when administered topically or systemically [91]. They regulate the immune response by increasing anti-inflammatory substances and inhibiting pro-inflammatory cytokines, which lowers inflammation and stops excessive scarring from forming. This double method of action enhances the results of burn injuries overall, as well as wound healing and tissue regeneration [92]. Combining bone marrow-derived mesenchymal stem cells (BMSCs) with platelet-rich plasma (PRP) has shown significant improvements in burn wound healing in rat models [93]. This therapy reduced burn area, faster epithelization, and increased burn contracture rate compared to traditional treatments. It also accelerated wound closure and re-epithelialization by enhancing epidermal cell proliferation and differentiation [93]. Angiogenesis, crucial for tissue repair, was promoted through increased expression of vascular endothelial growth factor. The combination therapy also improved scar regulating parameters, decreased oxidative stress, and modulated the inflammatory response [93, 94].

### Cancer treatment

The use of stem cell therapy in cancer treatment should be carefully discussed and considered by clinicians and researchers to ensure the safety and efficacy of the options. Some stem cell clinics often offer three types of SCTs for cancer treatment: autologous hematopoietic stem cell transplants, stromal vascular fraction, and multipotent stem cells like mesenchymal stem cells. Allogeneic HSCTs have demonstrated promise in producing donor lymphocytes capable of suppressing and regressing haematological malignancies and some solid tumours, a phenomenon known as the “graft-versus-tumor regression effect” [95]. However, scientific data does not support the safety and efficacy of allogeneic cell-based therapy for treating solid tumours. The Cochrane Library states that marrow transplantation with autologous HSCTs and high-dose chemotherapy does not increase overall survival in women with metastatic breast cancer [96]. Additionally, a study of over 41,000 breast cancer patients found no significant difference in survival benefits between those who had HSCTs following high-dose chemotherapy and those who received standard treatment [97].

Preclinical studies have highlighted the potential of MSC-based therapy in cancer treatment. MSCs can migrate to injured sites in response to growth factors, cytokines, and chemokines [98]. These cells express specific receptors, such as CXCR4 and CCR1-7, which aid in responding to environmental cues. MSCs also have adhesion molecules on their surface, such as CD49d, CD44, CD54, CD102, and CD106, which facilitate attachment, migration, and penetration of blood vessel lumens into injured tissue. Tumors, like injured tissues, generate chemoattractants that induce MSC migration via the CXCL12/CXCR4 axis [99]. Once MSCs migrate to malignant tissue, they can interact with cancer cells, demonstrating both protumor and antitumor effects, which are crucial considerations in MSC-based therapy. MSCs are known for their regenerative abilities, which promote tissue repair and recovery. They also have unique abilities that contribute to their protumor behavior. Studies have shown that breast cancer cells increase MSC production of chemokine (C-C motif) ligand 5 (CCL-5), which regulates tumour invasion [100]. MSCs also release growth factors such as VEGF, basic FGF, HGF, and PDGF, which can suppress cancer cell death. Despite their role in protumor activities, MSCs have significant tumor-suppressive properties that have been used in cancer treatment [99]. They can block the Wnt and AKT signalling pathways, decrease angiogenesis, increase inflammatory cell infiltration, and trigger tumor cell cycle arrest and apoptosis. However, the specific roles of MSCs in both protumor and anticancer actions remain debatable in the stem cell field [26, 101].

Numerous clinical trials have been profiled on ClinicalTrials.gov to examine the potential of mesenchymal stem cells (MSCs) as a cancer treatment. These trials, mostly phase 1 and 2 studies, aim to evaluate the safety and efficacy of MSC-based therapy [102]. Some researchers have investigated combining MSC-based therapy with an oncolytic viral approach, which involves using viruses that selectively infect and kill cancer cells while leaving healthy cells unharmed [103]. For example, a study by Rincón et al. (2017) utilised bone marrow-derived MSCs infected with the oncolytic adenovirus ICOVIR5 to treat metastatic and resistant solid tumours in both pediatric and adult patients. The study demonstrated the treatment’s safety and provided preliminary evidence of its therapeutic potential [104]. Another study published in 2019 showed that adipose-derived MSCs injected with the vaccinia virus could eradicate resistant tumor cells by enhancing virus amplification and sensitising tumour cells to virus infection [105].

### Diabetes

Since the pancreas has limited regenerative capacity for its islets, alternative cell sources are being explored in cases of failure. PSCs are highly regarded for their potential in beta cell replacement therapies. Several clinical trials are underway to replace beta cells using ESCs [106]. These trials involve implanting insulin-producing beta cells within an encapsulating device beneath the skin to protect them from autoimmune attacks in type 1 diabetes patients [107]. One example of such a trial is the phase I/II trial launched by ViaCyteTM in partnership with Harvard University in 2014, which involves 40 patients and uses two subcutaneous capsules containing insulin-producing beta cells generated from ESCs. Preclinical studies have shown successful blood sugar correction and viable, intact insulin-producing cells after 174 days [108].

Treating diabetic foot ulcers (DFUs) in Africa using SCT is a multifaceted process influenced by preclinical and clinical studies. BM-MSCs are typically preferred in clinical settings, with autologous cells being the preferred choice [96]. However, the choice of stem cell type remains controversial, with bone marrow and peripheral blood-derived MSCs also being used frequently [109]. Adipose-derived stem cells (ADSCs) are preferred due to their simpler isolation methods and promising outcomes. However, challenges such as metabolic changes and advanced age in DFU patients necessitate alternative approaches like allogeneic therapy [109]. Both local and systemic delivery routes have shown efficacy, with local injections being the most common method. Bioengineered products like Graftjacket and Epifix offer promising avenues for stem cell therapy delivery. Further research is needed to optimize the use of stem cell therapy for DFU treatment in Africa.

### Neurological disorders

SCT holds significant promise in the realm of neurological disorders, particularly in conditions like Alzheimer’s disease (AD), Parkinson’s disease (PD), multiple sclerosis (MS), glioblastoma multiforme (GBM), and ischemic stroke (IS).

### Alzheimer’s disease (AD)

MSCs can repair injured neurons, lessen cell death, and remove harmful aggregates linked to the pathophysiology of AD [44]. Research has demonstrated that in animal models of AD, MSC transplantation can lower Tau phosphorylation, enhance cognitive performance, and lessen inflammation. Additionally, increased neurogenesis following MSC transplantation has been linked to the amelioration of AD-like diseases. Furthermore, using MSC-derived exosomes loaded with miRNAs—like miR-29a and miR-21—has demonstrated encouraging outcomes in lowering Aβ levels and delaying the decline of cognitive function in AD models. The clinical trial (NCT05233774) has also shown enhanced cognition scores and increased hippocampus volume, indicating the safety and possible effectiveness of MSC transplantation in AD patients [110].

### Parkinson’s disease (PD)

MSCs have been reported to treat motor and non-motor symptoms in Parkinson’s disease. Through trophic activities fueled by cytokines, neuroprotective factors, and differentiation into unique cell types that aid in cell replenishment, MSC transplantation alters disease symptoms and development. Numerous MSC transplantation strategies, including autologous and allogeneic methods, as well as the application of the MSC secretome, have been investigated in studies [111]. To alter the course of the disease and manage its symptoms, clinical trials assessing the safety and effectiveness of MSC-based therapy in PD patients are now being conducted [112].

### Multiple sclerosis (MS)

MSCs and their exosomes have emerged as prospective treatment alternatives for MS given their capacity to lessen neuroinflammation, enhance remyelination, and ameliorate motor function deficiencies. Exosomes produced from MSCs have been demonstrated in studies to reduce proinflammatory responses and lessen demyelination in MS models. Clinical studies with the identifier NCT02166021 have looked into the effectiveness and safety of MSC transplantation in MS patients [113, 114]. According to this experiment, MSCs seem to be a promising therapy option for multiple sclerosis, with intrathecal application being the most effective mode of administration. Some studies have also shown improvements in neurological function and a decrease in disease activity [115].

### Glioblastoma Multiforme (GBM)

GBM’s aggressiveness and intricate tumor microenvironment provide special challenges. Studies have investigated the administration of anti-cancer medicines, including microRNAs and tumor necrosis factor-related apoptosis-inducing ligand (TRAIL), via MSCs, although care is advised due to their possible role in tumor progression. With an emphasis on tactics such as oncolytic virus-loaded MSCs and suicide gene-expressing MSCs, clinical trials (NCT03896568 and NCT04657315) are being conducted to assess the safety and effectiveness of MSC-based therapy in GBM patients [113, 116].

### Ischemic stroke (IS)

MSC transplantation has demonstrated potential for improving vascularization and neurogenesis, decreasing infarct volume, and accelerating neurological recovery in IS [116]. Research has looked into several delivery methods and cell sources, such as MSCs produced from bone marrow and umbilical cord, as well as the use of exosomes derived from MSCs that are loaded with neuroprotective microRNAs. Clinical investigations such as NCT01716481, NCT01461720, NCT04280003, NCT00875654, and NCT04280003 have all indicated improvements in motor function and biomarkers linked to neurogenesis and neuroplasticity, as well as the safety of MSC transplantation in IS patients [113].

### Recommendations

To enhance SCT in Africa, it is critical to build a strong regulatory framework that meets international standards. This framework should establish clear criteria for the derivation, processing, and clinical application of stem cells, as well as protect patients from fraudulent practices. Furthermore, there is an increasing need to engage in capacity building by offering financial incentives to dissuade qualified workers from migrating and boosting stem cell education to cultivate and keep talent in African countries. Efforts should be made to guarantee that SCT is affordable to the general population. This can be accomplished by providing government subsidies for medical insurance and advocating for the inclusion of SCT in various medical insurance policies. Addressing religious resistance is also critical; educational activities aimed at religious communities can debunk myths and promote informed decision-making regarding support for stem cell research. Extensive public education campaigns are required to improve knowledge of stem cells, their potential advantages, and their role in disease treatment, such as HIV/AIDS. These efforts should try to dispel myths, provide factual information, and demonstrate the practical benefits of SCT using examples and testimonies. Finally, showing the safety and efficacy of SCTs using empirical evidence might help ease scepticism and anxieties, eventually leading to greater acceptance of stem cells.

## Conclusion

In conclusion, SCT appears to be a promising path for improving health outcomes among Africans suffering from neurodegenerative illnesses and most non-communicable diseases. Despite significant progress made in animal studies and clinical trials, further refining is required to maximise cell therapy’s efficacy in the human body. Nonetheless, scientists’ major advances encourage confidence in SCT’s ability to tackle neurodegenerative disorders effectively. Continued devotion and innovation in this discipline present promising opportunities for improving many people’s lives impacted by these disorders. Furthermore, SCT is moving from a theoretical possibility to a physical reality, thanks to a decade of intense study. Despite ongoing hurdles, the discipline has made significant progress, with successful clinical studies for diseases such as neurodegenerative disorders and macular degeneration. Notably, iPSCs are revolutionising research by providing personalised treatment choices based on patients’ cells. Furthermore, the clinical use of MSCs to regenerate dental and periodontal tissues demonstrates the near-term viability of SCTs. Despite formidable challenges, rapid advancements in stem cell research pave the way for the widespread adoption of cellular therapeutics. Globally, concerted efforts are underway to establish regulatory guidelines and standards to ensure patient safety. The emergence of SCTs is poised to profoundly impact human health, offering new avenues for treating a myriad of debilitating conditions.

## Abbreviations

NCDs: Non-Communicable Diseases
NSCs: Neural Stem Cells
ESCs: Embryonic Stem Cells
IPSCs: Induced Pluripotent Stem Cells
MSCs: Mesenchymal Stem Cells
CVDs: Cardiovascular Diseases
HSCT: Hematopoietic Stem Cell Transplantation
CFUs: Colony-Forming Units
hESCs: Human Embryonic Stem Cells
ASCs: Adult Stem Cells
TE: Trophectoderm
ICM: Inner Cell Mass
USC: Urine-Derived Stem Cells
DSCs: Dental Stem Cells
MDSCs: Myogenic Stem Cells
IBRO-ARC: International Brain Research Organisation (IBRO)-African Regional Committee (ARC)
BNRN: Bone Marrow Registry, Nigeria
CAR-T: Chimeric Antigen Receptor T cell
LMICs: Low-Middle Income Countries
SCD: Sickle Cell Disease
WHO: World Health Organisation
CD: Crohn’s disease
IBD: Inflammatory Bowel Disease
NAFLD: Nonalcoholic Fatty Liver Disease
ALD: Autoimmune Liver Disease
ACLF: Acute-On-Chronic Liver Failure
UC-MSCs: Umbilical Cord-Derived MSCs
BM-MSCs: Bone Marrow-Derived MSCS
BMNCs: Bone Marrow Mononuclear Cells
PBC: Primary Biliary Cholangitis
UDCA: Ursodeoxycholic Acid
OA: Osteoarthritis
AT-MSCs: Adipose Tissue-Derived Mscs
CXCR4: Chemokine Receptor Type 4
PSCs: Pluripotent Stem Cells
TGF-β1: Tissue Growth Factor
PDGF: Platelets-Derived Growth Factor
FGF-2: Fibroblast Growth Factor
VEGF: Vascular Endothelial Growth Factor
DFU: Diabetic Foot Ulcer

## References

[1] Alatyyat SM, Alasmari HM, Aleid OA, Abdel-maksoud MS, Elsherbiny N. Umbilical cord stem cells: Background, processing and applications. Tissue Cell [Internet]. 2020;65:101351. https://linkinghub.elsevier.com/retrieve/pii/S0040816619305270 10.1016/j.tice.2020.101351 32746993

[2] Marks PW, Witten CM, Califf RM. Clarifying Stem-Cell Therapy’s Benefits and Risks. N Engl J Med [Internet]. 2017;376(11):1007–9. http://www.nejm.org/doi/10.1056/NEJMp1613723 10.1056/NEJMp1613723 27959704

[3] Zakrzewski W, Dobrzyński M, Szymonowicz M, Rybak Z. Stem cells: past, present, and future. Stem Cell Res Ther [Internet]. 2019;10(1):68. https://stemcellres.biomedcentral.com/articles/10.1186/s13287-019-1165-5 10.1186/s13287-019-1165-5 PMC6390367 30808416

[4] Jin Y, Li S, Yu Q, Chen T, Liu D. Application of stem cells in regeneration medicine. MedComm [Internet]. 2023;4(4). https://onlinelibrary.wiley.com/doi/10.1002/mco2.291 10.1002/mco2.291 PMC10276889 37337579

[5] Yamanaka S. Pluripotent Stem Cell-Based Cell Therapy—Promise and Challenges. Cell Stem Cell [Internet]. 2020;27(4):523–31. https://linkinghub.elsevier.com/retrieve/pii/S1934590920304604 10.1016/j.stem.2020.09.014 33007237

[6] Chen X, Tang H, Lin J, Zeng R. Temporal trends in the disease burden of osteoarthritis from 1990 to 2019, and projections until 2030. Sung WW, editor. PLoS One [Internet]. 2023;18(7):e0288561. 10.1371/journal.pone.0288561 10.1371/journal.pone.0288561 PMC10365297 37486949

[7] World Health Organization. Cardiovascular diseases (CVDs) [Internet]. 2021. https://www.who.int/news-room/fact-sheets/detail/cardiovascular-diseases-(cvds).

[8] Lampropoulos IC, Malli F, Sinani O, Gourgoulianis KI, Xiromerisiou G. Worldwide trends in mortality related to Parkinson’s disease in the period of 1994–2019: Analysis of vital registration data from the WHO Mortality Database. Front Neurol [Internet]. 2022;13. https://www.frontiersin.org/articles/10.3389/fneur.2022.956440/full 10.3389/fneur.2022.956440 PMC9576872 36267881

[9] Safiri S, Karamzad N, Kaufman JS, Bell AW, Nejadghaderi SA, Sullman MJM et al. Prevalence, Deaths and Disability-Adjusted-Life-Years (DALYs) Due to Type 2 Diabetes and Its Attributable Risk Factors in 204 Countries and Territories, 1990–2019: Results From the Global Burden of Disease Study 2019. Front Endocrinol (Lausanne) [Internet]. 2022;13. https://www.frontiersin.org/articles/10.3389/fendo.2022.838027/full 10.3389/fendo.2022.838027 PMC8915203 35282442

[10] World Health Organization. Burns [Internet]. 2023. https://www.who.int/news-room/fact-sheets/detail/burns

[11] World Health Organization. Spinal Cord Injury [Internet]. 2024. https://www.who.int/news-room/fact-sheets/detail/spinal-cord-injury

[12] Minja NW, Nakagaayi D, Aliku T, Zhang W, Ssinabulya I, Nabaale J et al. Cardiovascular diseases in Africa in the twenty-first century: Gaps and priorities going forward. Front Cardiovasc Med [Internet]. 2022;9. https://www.frontiersin.org/articles/10.3389/fcvm.2022.1008335/full PMC9686438 36440012

[13] Gowshall M, Taylor-Robinson S. The increasing prevalence of non-communicable diseases in low-middle income countries: the view from Malawi. Int J Gen Med [Internet]. 2018;Volume 11:255–64. https://www.dovepress.com/the-increasing-prevalence-of-non-communicable-diseases-in-low-middle-i-peer-reviewed-article-IJGM 10.2147/IJGM.S157987 PMC6029598 29988742

[14] Delobelle P, Sanders D, Puoane T, Freudenberg N. Reducing the Role of the Food, Tobacco, and Alcohol Industries in Noncommunicable Disease Risk in South Africa. Heal Educ Behav [Internet]. 2016;43(1_suppl):70S-81S. http://journals.sagepub.com/doi/10.1177/1090198115610568 10.1177/1090198115610568 27037150

[15] Sivandzade F, Cucullo L. Regenerative Stem Cell Therapy for Neurodegenerative Diseases: An Overview. Int J Mol Sci [Internet]. 2021;22(4):2153. https://www.mdpi.com/1422-0067/22/4/2153 10.3390/ijms22042153 PMC7926761 33671500

[16] Li X, Feng X, Sun X, Hou N, Han F, Liu Y. Global, regional, and national burden of Alzheimer’s disease and other dementias, 1990–2019. Front Aging Neurosci [Internet]. 2022;14. https://www.frontiersin.org/articles/10.3389/fnagi.2022.937486/full 10.3389/fnagi.2022.937486 PMC9588915 36299608

[17] Ramalho-Santos M, Willenbring H. On the Origin of the Term Stem Cell. Cell Stem Cell [Internet]. 2007;1(1):35–8. https://linkinghub.elsevier.com/retrieve/pii/S1934590907000197 10.1016/j.stem.2007.05.013 18371332

[18] Konstantinov IE. In Search of Alexander A., Maximow. The Man Behind the Unitarian Theory of Hematopoiesis. Perspect Biol Med [Internet]. 2000;43(2):269–76. https://muse.jhu.edu/article/46501 10.1353/pbm.2000.0006 10804590

[19] Cooper B. The origins of Bone Marrow as the Seedbed of our blood: from antiquity to the time of Osler. Bayl Univ Med Cent Proc. 2011;24(2):115–8. 10.1080/08998280.2011.11928697 PMC3069519 21566758

[20] Ramalho-Santos M, Willenbring H. On the origin of the term stem cell. Cell Stem Cell. 2007;1(1):35–8. 18371332 10.1016/j.stem.2007.05.013

[21] Dröscher A. Images of cell trees, cell lines, and cell fates: the legacy of Ernst Haeckel and August Weismann in stem cell research. Hist Philos Life Sci [Internet]. 2014;36(2):157–86. http://link.springer.com/10.1007/s40656-014-0028-8 10.1007/s40656-014-0028-8 25515356

[22] Jansen J. The First Successful Allogeneic Bone-Marrow Transplant: Georges Mathé. Transfus Med Rev [Internet]. 2005;19(3):246–8. https://linkinghub.elsevier.com/retrieve/pii/S0887796305000180 10.1016/j.tmrv.2005.02.006 16010655

[23] Blume KG, Weissman ILE, Donnall Thomas. (1920–2012). Proc Natl Acad Sci [Internet]. 2012;109(51):20777–8. 10.1073/pnas.1218913109 10.1073/pnas.1218913109 PMC3529056 23197829

[24] Cheng M. H artmann < scp > S tahelin (1925–2011) and the contested history of cyclosporin < scp > A. Clin Transplant [Internet]. 2013;27(3):326–9. https://onlinelibrary.wiley.com/doi/10.1111/ctr.12072 10.1111/ctr.12072 23331048

[25] Donnall Thomas E, Storb R, Fefer A, Slichter S, Bryant J, Dean Buckner C, APLASTIC ANAEMIA TREATED BY MARROW TRANSPLANTATION. Lancet [Internet]. 1972;299(7745):284–9. https://linkinghub.elsevier.com/retrieve/pii/S0140673672902929 10.1016/s0140-6736(72)90292-9 4109931

[26] Liang W, Chen X, Zhang S, Fang J, Chen M, Xu Y et al. Mesenchymal stem cells as a double-edged sword in tumor growth: focusing on MSC-derived cytokines. Cell Mol Biol Lett [Internet]. 2021;26(1):3. https://cmbl.biomedcentral.com/articles/10.1186/s11658-020-00246-5 10.1186/s11658-020-00246-5 PMC7818947 33472580

[27] Charitos IA, Ballini A, Cantore S, Boccellino M, Di Domenico M, Borsani E et al. Stem Cells: A Historical Review about Biological, Religious, and Ethical Issues. Mascitti M, editor. Stem Cells Int [Internet]. 2021;2021:1–11. https://www.hindawi.com/journals/sci/2021/9978837/10.1155/2021/9978837 PMC8105090 34012469

[28] Hoang DM, Pham PT, Bach TQ, Ngo ATL, Nguyen QT, Phan TTK et al. Stem cell-based therapy for human diseases. Signal Transduct Target Ther [Internet]. 2022;7(1):272. https://www.nature.com/articles/s41392-022-01134-4 10.1038/s41392-022-01134-4 PMC9357075 35933430

[29] Thomson JA, Itskovitz-Eldor J, Shapiro SS, Waknitz MA, Swiergiel JJ, Marshall VS et al. Embryonic Stem Cell Lines Derived from Human Blastocysts. Science (80-) [Internet]. 1998;282(5391):1145–7. https://www.science.org/doi/10.1126/science.282.5391.1145 10.1126/science.282.5391.1145 9804556

[30] Ballen KK, Logan BR, Laughlin MJ, He W, Ambruso DR, Armitage SE et al. Effect of Cord Blood Processing on Transplantation Outcomes after Single Myeloablative Umbilical Cord Blood Transplantation. Biol Blood Marrow Transplant [Internet]. 2015;21(4):688–95. https://linkinghub.elsevier.com/retrieve/pii/S1083879114014360 10.1016/j.bbmt.2014.12.017 PMC4359657 25543094

[31] Bolli R, Tang XL, Guo Y, Li Q. After the storm: an objective appraisal of the efficacy of c-kit + cardiac progenitor cells in preclinical models of heart disease. Can J Physiol Pharmacol [Internet]. 2021;99(2):129–39. 10.1139/cjpp-2020-0406 10.1139/cjpp-2020-0406 PMC8299902 32937086

[32] De D, Karmakar P, Bhattacharya D. Stem Cell Aging and Regenerative Medicine. In. 2020. pp. 11–37. https://link.springer.com/10.1007/5584_2020_577 10.1007/5584_2020_577 32910426

[33] Li W, Li L, Cui R, Chen X, Hu H, Qiu Y. Bone marrow mesenchymal stem cells derived exosomal Lnc TUG1 promotes bone fracture recovery via miR-22-5p/Anxa8 axis. Hum Cell [Internet]. 2023;36(3):1041–53. https://link.springer.com/10.1007/s13577-023-00881-y 10.1007/s13577-023-00881-y PMC10110643 36952210

[34] Yin X, Li Q, McNutt PM, Zhang Y. Urine-Derived Stem Cells for Epithelial Tissues Reconstruction and Wound Healing. Pharmaceutics [Internet]. 2022;14(8):1669. https://www.mdpi.com/1999-4923/14/8/1669 10.3390/pharmaceutics14081669 PMC9415563 36015295

[35] Freitag J, Bates D, Wickham J, Shah K, Huguenin L, Tenen A et al. Adipose-Derived Mesenchymal Stem Cell Therapy in the Treatment of Knee Osteoarthritis: A Randomized Controlled Trial. Regen Med [Internet]. 2019;14(3):213–30. https://www.tandfonline.com/doi/full/10.2217/rme-2018-0161 10.2217/rme-2018-0161 30762487

[36] Conese M, Annacontini L, Carbone A, Beccia E, Cecchino LR, Parisi D et al. The Role of Adipose-Derived Stem Cells, Dermal Regenerative Templates, and Platelet-Rich Plasma in Tissue Engineering-Based Treatments of Chronic Skin Wounds. Stem Cells Int [Internet]. 2020;2020:1–17. https://www.hindawi.com/journals/sci/2020/7056261/10.1155/2020/7056261 PMC7199611 32399048

[37] Herreros MD, Ramirez JM, Otero-Piñeiro AM, Martí-Gallostra M, Badiola I, Enríquez-Navascues JM et al. Use of Darvadstrocel (Allogenic Stem Cell Therapy) for Crohn’s Fistulas in Real Clinical Practice: The National Project to Implement Mesenchymal Stem Cell for the Treatment of Perianal Crohn’s Fistula (the PRIME Study). Dis Colon Rectum [Internet]. 2024;67(7):960–7. https://journals.lww.com/10.1097/DCR.0000000000003216 10.1097/DCR.0000000000003216 38603800

[38] Galindo S, de la Mata A, López-Paniagua M, Herreras JM, Pérez I, Calonge M et al. Subconjunctival injection of mesenchymal stem cells for corneal failure due to limbal stem cell deficiency: state of the art. Stem Cell Res Ther [Internet]. 2021;12(1):60. https://stemcellres.biomedcentral.com/articles/10.1186/s13287-020-02129-0 10.1186/s13287-020-02129-0 PMC7805216 33441175

[39] Benny M, Courchia B, Shrager S, Sharma M, Chen P, Duara J et al. Comparative Effects of Bone Marrow-derived Versus Umbilical Cord Tissue Mesenchymal Stem Cells in an Experimental Model of Bronchopulmonary Dysplasia. Stem Cells Transl Med [Internet]. 2022;11(2):189–99. https://academic.oup.com/stcltm/article/11/2/189/6542874 10.1093/stcltm/szab011 PMC8929420 35298658

[40] Ahn SY, Chang YS, Kim JH, Sung SI, Park WS. Two-Year Follow-Up Outcomes of Premature Infants Enrolled in the Phase I Trial of Mesenchymal Stem Cells Transplantation for Bronchopulmonary Dysplasia. J Pediatr [Internet]. 2017;185:49–54.e2. https://linkinghub.elsevier.com/retrieve/pii/S002234761730327X 10.1016/j.jpeds.2017.02.061 28341525

[41] Cruz FF, Rocco PRM. The potential of mesenchymal stem cell therapy for chronic lung disease. Expert Rev Respir Med [Internet]. 2020;14(1):31–9. https://www.tandfonline.com/doi/full/10.1080/17476348.2020.1679628 10.1080/17476348.2020.1679628 31608724

[42] Özgül Özdemir RB, Özdemir AT, Kırmaz C, Ovalı E, Ölmez E, Kerem H et al. Mesenchymal Stem Cells: a Potential Treatment Approach for Refractory Chronic Spontaneous Urticaria. Stem Cell Rev Reports [Internet]. 2021;17(3):911–22. https://link.springer.com/10.1007/s12015-020-10059-w 10.1007/s12015-020-10059-w 33089453

[43] Azeez IA, Awogbindin IO, Olayinka JN, Folarin RO, Adamu AS, Ior LD et al. Neural stem cell research in Africa: current realities and future prospects. Biol Open [Internet]. 2022;11(11). https://journals.biologists.com/bio/article/11/11/bio059574/280534/Neural-stem-cell-research-in-Africa-current 10.1242/bio.059574 PMC9641530 36326097

[44] Cecerska-Heryć E, Pękała M, Serwin N, Gliźniewicz M, Grygorcewicz B, Michalczyk A et al. The Use of Stem Cells as a Potential Treatment Method for Selected Neurodegenerative Diseases: Review. Cell Mol Neurobiol [Internet]. 2023;43(6):2643–73. https://link.springer.com/10.1007/s10571-023-01344-6 10.1007/s10571-023-01344-6 PMC10333383 37027074

[45] Ballo R, Greenberg LJ, Kidson SH. A New Class of Stem Cells in South Africa: Introducing Induced Pluripotent Stem cells (iPS cells). South African Med J [Internet]. 2012;103(1):16. http://www.samj.org.za/index.php/samj/article/view/6604 10.7196/samj.6604 23237114

[46] Mahmoud HK, Fathy GM, Elhaddad A, Fahmy OA, Abdelmooti M, Abdelfattah R, HEMATOPOIETIC STEM CELL TRANSPLANTATION IN EGYPT CHALLENGES AND SOLUTIONS. Mediterr J Hematol Infect Dis [Internet]. 2020;12(1):e2020023. http://mjhid.org/index.php/mjhid/article/view/2020.023 10.4084/MJHID.2020.023 PMC7202352 32395212

[47] Benchekroun S, Harif M, Madani A, Quessar A, Zafad S, Rachid R. Present and future of hematology and stem cell transplantation in Morocco. Bone Marrow Transplant [Internet]. 2008;42(S1):S106–8. https://www.nature.com/articles/bmt2008130 10.1038/bmt.2008.345 PMC7104431 18724279

[48] Bucher C. First successful allogeneic hematopoietic stem cell transplantation for a sickle cell disease patient in a low resource country (Nigeria): A case report. Ann Transplant [Internet]. 2014;19:210–3. http://www.annalsoftransplantation.com/abstract/index/idArt/890248 10.12659/AOT.890248 24792997

[49] Kirby T. Nigeria’s bone marrow registry offers new hope to patients. Lancet [Internet]. 2012;379(9832):2138. https://linkinghub.elsevier.com/retrieve/pii/S0140673612609295 10.1016/s0140-6736(12)60929-5 22690395

[50] Chen YS. Mesenchymal Stem Cell: Considerations for Manufacturing and Clinical Trials on Cell Therapy Product. Int J Stem cell Res Ther [Internet]. 2016;3(1). https://clinmedjournals.org/articles/ijscrt/international-journal-of-stem-cell-research-and-therapy-ijscrt-3-029.php?jid=ijscrt

[51] Gaobotse G. Stem Cell Research in Africa: Legislation and Challenges. J Regen Med [Internet]. 2018;07(01). https://www.scitechnol.com/peer-review/stem-cell-research-in-africa-legislation-and-challenges-1E3H.php?article_id=7753

[52] Maina MB, Ahmad U, Ibrahim HA, Hamidu SK, Nasr FE, Salihu AT et al. Two decades of neuroscience publication trends in Africa. Nat Commun [Internet]. 2021;12(1):3429. https://www.nature.com/articles/s41467-021-23784-8 10.1038/s41467-021-23784-8 PMC8187719 34103514

[53] Daniel FM, Essien EA, Gbuchie MA, Ukoaka BM, Emeruwa VE. Mitigating Physician Emigration in Nigeria by Improving the Internship Experience. Int J Med Students [Internet]. 2023;11(4):343–6. https://ijms.info/IJMS/article/view/2255

[54] Jackson CS, Pepper MS. Opportunities and barriers to establishing a cell therapy programme in South Africa. Stem Cell Res Ther [Internet]. 2013;4(5):54. https://stemcellres.biomedcentral.com/articles/10.1186/scrt204 10.1186/scrt204 PMC3707026 23719318

[55] Cornetta K, Bonamino M, Mahlangu J, Mingozzi F, Rangarajan S, Rao J. Gene therapy access: Global challenges, opportunities, and views from Brazil, South Africa, and India. Mol Ther [Internet]. 2022;30(6):2122–9. https://linkinghub.elsevier.com/retrieve/pii/S1525001622002301 10.1016/j.ymthe.2022.04.002 PMC9171243 35390542

[56] Senior M. After Glybera’s withdrawal, what’s next for gene therapy? Nat Biotechnol [Internet]. 2017;35(6):491–2. https://www.nature.com/articles/nbt0617-491 10.1038/nbt0617-491 28591128

[57] Yadav H, Shah D, Sayed S, Horton S, Schroeder LF. Availability of essential diagnostics in ten low-income and middle-income countries: results from national health facility surveys. Lancet Glob Heal [Internet]. 2021;9(11):e1553–60. https://linkinghub.elsevier.com/retrieve/pii/S2214109X21004423 10.1016/S2214-109X(21)00442-3 PMC8526361 34626546

[58] Pierz AJ, Randall TC, Castle PE, Adedimeji A, Ingabire C, Kubwimana G et al. A scoping review: Facilitators and barriers of cervical cancer screening and early diagnosis of breast cancer in Sub-Saharan African health settings. Gynecol Oncol Reports [Internet]. 2020;33:100605. https://linkinghub.elsevier.com/retrieve/pii/S2352578920300710 10.1016/j.gore.2020.100605 PMC7327246 32637528

[59] Cashin C, Dossou JP. Can National Health Insurance Pave the Way to Universal Health Coverage in Sub-Saharan Africa? Heal Syst Reform [Internet]. 2021;7(1). https://www.tandfonline.com/doi/full/10.1080/23288604.2021.2006122 10.1080/23288604.2021.2006122 34965364

[60] Amarachukwu CN, Okoronkwo IL, Nweke MC, Ukwuoma MK. Economic burden and catastrophic cost among people living with sickle cell disease, attending a tertiary health institution in south-east zone, Nigeria. Hodges MH, editor. PLoS One [Internet]. 2022;17(8):e0272491. 10.1371/journal.pone.0272491 10.1371/journal.pone.0272491 PMC9398014 35998131

[61] Adair JE, Androski L, Bayigga L, Bazira D, Brandon E, Dee L et al. Towards access for all: 1st Working Group Report for the Global Gene Therapy Initiative (GGTI). Gene Ther [Internet]. 2023;30(3–4):216–21. https://www.nature.com/articles/s41434-021-00284-4 10.1038/s41434-021-00284-4 PMC10113145 34493840

[62] Munung NS, Nnodu OE, Moru PO, Kalu AA, Impouma B, Treadwell MJ et al. Looking ahead: ethical and social challenges of somatic gene therapy for sickle cell disease in Africa. Gene Ther [Internet]. 2023; https://www.nature.com/articles/s41434-023-00429-7 10.1038/s41434-023-00429-7 PMC11090833 38012299

[63] Rzymski P, Szuster-Ciesielska A, Dzieciątkowski T, Gwenzi W, Fal A. mRNA vaccines: The future of prevention of viral infections? J Med Virol [Internet]. 2023;95(2). https://onlinelibrary.wiley.com/doi/10.1002/jmv.28572 10.1002/jmv.28572 36762592

[64] Zhang J, Bolli R, Garry DJ, Marbán E, Menasché P, Zimmermann WH et al. Basic and Translational Research in Cardiac Repair and Regeneration. J Am Coll Cardiol [Internet]. 2021;78(21):2092–105. https://linkinghub.elsevier.com/retrieve/pii/S0735109721063208 10.1016/j.jacc.2021.09.019 PMC9116459 34794691

[65] Hou J, Jiang T, Fu J, Su B, Wu H, Sun R et al. The Long-Term Efficacy of Working Memory Training in Healthy Older Adults: A Systematic Review and Meta-Analysis of 22 Randomized Controlled Trials. Gutchess A, editor. Journals Gerontol Ser B [Internet]. 2020;75(8):e174–88. https://academic.oup.com/psychsocgerontology/article/75/8/e174/5854377 10.1093/geronb/gbaa077 32507890

[66] Gyöngyösi M, Wojakowski W, Navarese EP, Moye LÀ. Meta-Analyses of Human Cell-Based Cardiac Regeneration Therapies. Circ Res [Internet]. 2016;118(8):1254–63. https://www.ahajournals.org/doi/10.1161/CIRCRESAHA.115.307347 10.1161/CIRCRESAHA.115.307347 PMC4834852 27081108

[67] Gehart H, Clevers H. Tales from the crypt: new insights into intestinal stem cells. Nat Rev Gastroenterol Hepatol [Internet]. 2019;16(1):19–34. https://www.nature.com/articles/s41575-018-0081-y 10.1038/s41575-018-0081-y 30429586

[68] Santos AJM, Lo YH, Mah AT, Kuo CJ. The Intestinal Stem Cell Niche: Homeostasis and Adaptations. Trends Cell Biol [Internet]. 2018;28(12):1062–78. https://linkinghub.elsevier.com/retrieve/pii/S0962892418301399 10.1016/j.tcb.2018.08.001 PMC6338454 30195922

[69] Roda G, Chien Ng S, Kotze PG, Argollo M, Panaccione R, Spinelli A et al. Crohn’s disease. Nat Rev Dis Prim [Internet]. 2020;6(1):22. https://www.nature.com/articles/s41572-020-0156-2 10.1038/s41572-020-0156-2 32242028

[70] Kobayashi T, Siegmund B, Le Berre C, Wei SC, Ferrante M, Shen B et al. Ulcerative colitis. Nat Rev Dis Prim [Internet]. 2020;6(1):74. https://www.nature.com/articles/s41572-020-0205-x 10.1038/s41572-020-0205-x 32913180

[71] Lindsay JO, Allez M, Clark M, Labopin M, Ricart E, Rogler G et al. Autologous stem-cell transplantation in treatment-refractory Crohn’s disease: an analysis of pooled data from the ASTIC trial. Lancet Gastroenterol Hepatol [Internet]. 2017;2(6):399–406. https://linkinghub.elsevier.com/retrieve/pii/S2468125317300560 10.1016/S2468-1253(17)30056-0 28497755

[72] Wang R, Yao Q, Chen W, Gao F, Li P, Wu J et al. Stem cell therapy for Crohn’s disease: systematic review and meta-analysis of preclinical and clinical studies. Stem Cell Res Ther [Internet]. 2021;12(1):463. https://stemcellres.biomedcentral.com/articles/10.1186/s13287-021-02533-0 10.1186/s13287-021-02533-0 PMC8375136 34407875

[73] Hawkey CJ. Hematopoietic Stem Cell Transplantation in Crohn’s Disease: State-of-the-Art Treatment. Dig Dis [Internet]. 2017;35(1–2):107–14. https://www.karger.com/Article/FullText/449090 10.1159/000449090 28147358

[74] Si-Tayeb K, Lemaigre FP, Duncan SA. Organogenesis and Development of the Liver. Dev Cell [Internet]. 2010;18(2):175–89. https://linkinghub.elsevier.com/retrieve/pii/S1534580710000559 10.1016/j.devcel.2010.01.011 20159590

[75] Xue R, Meng Q, Dong J, Li J, Yao Q, Zhu Y et al. Clinical performance of stem cell therapy in patients with acute-on-chronic liver failure: a systematic review and meta-analysis. J Transl Med [Internet]. 2018;16(1):126. https://translational-medicine.biomedcentral.com/articles/10.1186/s12967-018-1464-0 10.1186/s12967-018-1464-0 PMC5946490 29747694

[76] Liu Y, Dong Y, Wu X, Xu X, Niu J. The assessment of mesenchymal stem cells therapy in acute on chronic liver failure and chronic liver disease: a systematic review and meta-analysis of randomized controlled clinical trials. Stem Cell Res Ther [Internet]. 2022;13(1):204. https://stemcellres.biomedcentral.com/articles/10.1186/s13287-022-02882-4 10.1186/s13287-022-02882-4 PMC9109309 35578365

[77] Lin B, Chen J, Qiu W, Wang K, Xie D, Chen X et al. Allogeneic bone marrow–derived mesenchymal stromal cells for hepatitis B virus–related acute-on‐chronic liver failure: A randomized controlled trial. Hepatology [Internet]. 2017;66(1):209–19. https://journals.lww.com/01515467-201707000-00020 10.1002/hep.29189 28370357

[78] Arroyo V, Moreau R, Kamath PS, Jalan R, Ginès P, Nevens F et al. Acute-on-chronic liver failure in cirrhosis. Nat Rev Dis Prim [Internet]. 2016;2(1):16041. https://www.nature.com/articles/nrdp201641 10.1038/nrdp.2016.41 27277335

[79] Zhang Z, Lin H, Shi M, Xu R, Fu J, Lv J et al. Human umbilical cord mesenchymal stem cells improve liver function and ascites in decompensated liver cirrhosis patients. J Gastroenterol Hepatol [Internet]. 2012;27(s2):112–20. https://onlinelibrary.wiley.com/doi/10.1111/j.1440-1746.2011.07024.x 10.1111/j.1440-1746.2011.07024.x 22320928

[80] Nguyen TL, Nguyen HP, Ngo DM, Ha THT, Mai KA, Bui TH et al. Autologous bone marrow mononuclear cell infusion for liver cirrhosis after the Kasai operation in children with biliary atresia. Stem Cell Res Ther [Internet]. 2022;13(1):108. https://stemcellres.biomedcentral.com/articles/10.1186/s13287-022-02762-x 10.1186/s13287-022-02762-x PMC8919575 35287722

[81] Spahr L, Chalandon Y, Terraz S, Kindler V, Rubbia-Brandt L, Frossard JL et al. Autologous Bone Marrow Mononuclear Cell Transplantation in Patients with Decompensated Alcoholic Liver Disease: A Randomized Controlled Trial. Gluud LL, editor. PLoS One [Internet]. 2013;8(1):e53719. 10.1371/journal.pone.0053719 10.1371/journal.pone.0053719 PMC3544843 23341981

[82] Huang T (Dazhong), Behary J, Zekry A, editors. Non-alcoholic fatty liver disease: a review of epidemiology, risk factors, diagnosis and management. Intern Med J [Internet]. 2020;50(9):1038–47. https://onlinelibrary.wiley.com/doi/10.1111/imj.14709 10.1111/imj.14709 31760676

[83] Sakai Y, Fukunishi S, Takamura M, Kawaguchi K, Inoue O, Usui S et al. Clinical trial of autologous adipose tissue-derived regenerative (stem) cells therapy for exploration of its safety and efficacy. Regen Ther [Internet]. 2021;18:97–101. https://linkinghub.elsevier.com/retrieve/pii/S2352320421000316 10.1016/j.reth.2021.04.003 PMC8165289 34095367

[84] Wang L, Li J, Liu H, Li Y, Fu J, Sun Y et al. A pilot study of umbilical cord-derived mesenchymal stem cell transfusion in patients with primary biliary cirrhosis. J Gastroenterol Hepatol [Internet]. 2013;28(S1):85–92. https://onlinelibrary.wiley.com/doi/10.1111/jgh.12029 10.1111/jgh.12029 23855301

[85] Martel-Pelletier J, Barr AJ, Cicuttini FM, Conaghan PG, Cooper C, Goldring MB et al. Osteoarthritis. Nat Rev Dis Prim [Internet]. 2016;2(1):16072. https://www.nature.com/articles/nrdp201672 10.1038/nrdp.2016.72 27734845

[86] Mahmoudian A, Lohmander LS, Mobasheri A, Englund M, Luyten FP. Early-stage symptomatic osteoarthritis of the knee — time for action. Nat Rev Rheumatol [Internet]. 2021;17(10):621–32. https://www.nature.com/articles/s41584-021-00673-4 10.1038/s41584-021-00673-4 34465902

[87] Kubsik-Gidlewska A, Klupiński K, Krochmalski M, Krochmalski J, Klimkiewicz P, Woldańska-Okońska M. CD34 + stem cell treatment for knee osteoarthritis: a treatment and rehabilitation algorithm. J Rehabil Med – Clin Commun [Internet]. 2018;1(1):1000012. https://medicaljournalssweden.se/jrm-cc/article/view/2614 10.2340/20030711-1000012 PMC8011677 33884126

[88] Jevotovsky DS, Alfonso AR, Einhorn TA, Chiu ES. Osteoarthritis and stem cell therapy in humans: a systematic review. Osteoarthr Cartil [Internet]. 2018;26(6):711–29. https://linkinghub.elsevier.com/retrieve/pii/S106345841831080X 10.1016/j.joca.2018.02.906 29544858

[89] Wiggers TG, Winters M, Van den Boom NA, Haisma HJ, Moen MH. Autologous stem cell therapy in knee osteoarthritis: a systematic review of randomised controlled trials. Br J Sports Med [Internet]. 2021;55(20):1161–9. https://bjsm.bmj.com/lookup/doi/10.1136/bjsports-2020-103671 10.1136/bjsports-2020-103671 34039582

[90] Han SB, Seo IW, Shin YS. Intra-Articular Injections of Hyaluronic Acid or Steroids Associated With Better Outcomes Than Platelet-Rich Plasma, Adipose Mesenchymal Stromal Cells, or Placebo in Knee Osteoarthritis: A Network Meta-analysis. Arthrosc J Arthrosc Relat Surg [Internet]. 2021;37(1):292–306. https://linkinghub.elsevier.com/retrieve/pii/S0749806320303182 10.1016/j.arthro.2020.03.041 32305424

[91] Otero-Viñas M, Falanga V. Mesenchymal Stem Cells in Chronic Wounds: The Spectrum from Basic to Advanced Therapy. Adv Wound Care [Internet]. 2016;5(4):149–63. http://www.liebertpub.com/doi/10.1089/wound.2015.0627 10.1089/wound.2015.0627 PMC4817558 27076993

[92] Soliman AM, Barreda DR. Acute Inflammation in Tissue Healing. Int J Mol Sci [Internet]. 2022;24(1):641. https://www.mdpi.com/1422-0067/24/1/641 10.3390/ijms24010641 PMC9820461 36614083

[93] Tammam BMH, Habotta OA, El-khadragy M, Abdel Moneim AE, Abdalla MS. Therapeutic role of mesenchymal stem cells and platelet-rich plasma on skin burn healing and rejuvenation: A focus on scar regulation, oxido-inflammatory stress and apoptotic mechanisms. Heliyon [Internet]. 2023;9(9):e19452. https://linkinghub.elsevier.com/retrieve/pii/S2405844023066604 10.1016/j.heliyon.2023.e19452 PMC10472052 37662797

[94] biomedicines-12-00743.pdf.crdownload.

[95] Barisic S, Childs RW. Graft-Versus-Solid-Tumor Effect: From Hematopoietic Stem Cell Transplantation to Adoptive Cell Therapies. Stem Cells [Internet]. 2022;40(6):556–63. https://academic.oup.com/stmcls/article/40/6/556/6553195 10.1093/stmcls/sxac021 PMC9216497 35325242

[96] Karadurmus N, Sahin U, Bahadir Basgoz B, Arpaci F, Demirer T. A Review of Allogeneic Hematopoietic Stem Cell Transplantation in Metastatic Breast Cancer. Int J Hematol stem cell Res [Internet]. 2018;12(2):111–6. http://www.ncbi.nlm.nih.gov/pubmed/30233772 PMC6141428 30233772

[97] Mello MM, Brennan TA, The Controversy Over High-Dose Chemotherapy With Autologous Bone Marrow Transplant For Breast Cancer. Health Aff [Internet]. 2001;20(5):101–17. http://www.healthaffairs.org/doi/10.1377/hlthaff.20.5.101 10.1377/hlthaff.20.5.101 11558695

[98] Sissung TM, Figg WD. Stem cell clinics: risk of proliferation. Lancet Oncol [Internet]. 2020;21(2):205–6. https://linkinghub.elsevier.com/retrieve/pii/S1470204519307879 10.1016/S1470-2045(19)30787-9 32007195

[99] Ding W, Knox TR, Tschumper RC, Wu W, Schwager SM, Boysen JC et al. Platelet-derived growth factor (PDGF)–PDGF receptor interaction activates bone marrow–derived mesenchymal stromal cells derived from chronic lymphocytic leukemia: implications for an angiogenic switch. Blood [Internet]. 2010;116(16):2984–93. https://ashpublications.org/blood/article/116/16/2984/27822/Plateletderived-growth-factor-PDGFPDGF-receptor 10.1182/blood-2010-02-269894 PMC2974606 20606160

[100] Aldinucci D, Borghese C, Casagrande N. The CCL5/CCR5 Axis in Cancer Progression. Cancers (Basel) [Internet]. 2020;12(7):1765. https://www.mdpi.com/2072-6694/12/7/1765 10.3390/cancers12071765 PMC7407580 32630699

[101] Rhee KJ, Lee J, Eom Y. Mesenchymal Stem Cell-Mediated Effects of Tumor Support or Suppression. Int J Mol Sci [Internet]. 2015;16(12):30015–33. http://www.mdpi.com/1422-0067/16/12/26215 10.3390/ijms161226215 PMC4691158 26694366

[102] Hmadcha A, Martin-Montalvo A, Gauthier BR, Soria B, Capilla-Gonzalez V. Therapeutic Potential of Mesenchymal Stem Cells for Cancer Therapy. Front Bioeng Biotechnol [Internet]. 2020;8. https://www.frontiersin.org/article/10.3389/fbioe.2020.00043/full 10.3389/fbioe.2020.00043 PMC7013101 32117924

[103] Cao Gdong, He X bo, Sun Q, Chen S, Wan K, Xu X et al. The Oncolytic Virus in Cancer Diagnosis and Treatment. Front Oncol [Internet]. 2020;10. https://www.frontiersin.org/articles/10.3389/fonc.2020.01786/full 10.3389/fonc.2020.01786 PMC7509414 33014876

[104] Rincón E, Cejalvo T, Kanojia D, Alfranca A, Rodríguez-Milla M. Ángel, Gil Hoyos R. Andrés, et al. Mesenchymal stem cell carriers enhance antitumor efficacy of oncolytic adenoviruses in an immunocompetent mouse model. Oncotarget. 2017;8:45415–31. Available from: https://www.oncotarget.com/article/17557/text/. 10.18632/oncotarget.17557 PMC5542197 28525366

[105] Draganov DD, Santidrian AF, Minev I, Nguyen D, Kilinc MO, Petrov I, et al. Delivery of oncolytic vaccinia virus by matched allogeneic stem cells overcomes critical innate and adaptive immune barriers. J Transl Med [Internet]. 2019;17(1):100. Available from: https://translational-medicine.biomedcentral.com/articles/10.1186/s12967-019-1829-z. 10.1186/s12967-019-1829-z PMC6437877 30917829

[106] Pagliuca FW, Millman JR, Gürtler M, Segel M, Van Dervort A, Ryu JH, et al. Generation of functional human pancreatic β cells in vitro. Cell [Internet]. 2014;159(2):428–39. Available from: https://linkinghub.elsevier.com/retrieve/pii/S0092867414012288. 10.1016/j.cell.2014.09.040 PMC4617632 25303535

[107] Schulz TC, Young HY, Agulnick AD, Babin MJ, Baetge EE, Bang AG, et al. A scalable system for production of functional pancreatic progenitors from human embryonic stem cells. Lynn FC, editor. PLoS One [Internet]. 2012;7(5):e37004. Available from: https://dx.plos.org/10.1371/journal.pone.0037004. 10.1371/journal.pone.0037004 PMC3356395 22623968

[108] Vegas AJ, Veiseh O, Gürtler M, Millman JR, Pagliuca FW, Bader AR, et al. Long-term glycemic control using polymer-encapsulated human stem cell–derived beta cells in immune-competent mice. Nat Med [Internet]. 2016;22(3):306–11. Available from: https://www.nature.com/articles/nm.4030. 10.1038/nm.4030 PMC4825868 26808346

[109] Lopes L, Setia O, Aurshina A, Liu S, Hu H, Isaji T, et al. Stem cell therapy for diabetic foot ulcers: a review of preclinical and clinical research. Stem Cell Res Ther [Internet]. 2018;9(1):188. Available from: https://stemcellres.biomedcentral.com/articles/10.1186/s13287-018-0938-6. 10.1186/s13287-018-0938-6 PMC6042254 29996912

[110] Horgan D, Nobili F, Teunissen C, Grimmer T, Mitrecic D, Ris L, et al. Biomarker testing: piercing the fog of alzheimer’s and related dementia. Biomed Hub [Internet]. 2020;5(3):1–22. Available from: https://karger.com/BMH/article/doi/10.1159/000511233. 10.1159/000511233 PMC7841748 33564663

[111] Berlet R, Galang Cabantan DA, Gonzales-Portillo D, Borlongan C V. Enriched environment and exercise enhance stem cell therapy for stroke, parkinson’s disease, and huntington’s disease. Front Cell Dev Biol [Internet]. 2022;10. Available from: https://www.frontiersin.org/articles/10.3389/fcell.2022.798826/full. 10.3389/fcell.2022.798826 PMC8927702 35309929

[112] Liu S fen, Li L yi, Zhuang J long, Li M mi, Ye L chao, Chen X rong, et al. Update on the application of mesenchymal stem cell-derived exosomes in the treatment of Parkinson’s disease: a systematic review. Front Neurol [Internet]. 2022;13. Available from: https://www.frontiersin.org/articles/10.3389/fneur.2022.950715/full. 10.3389/fneur.2022.950715 PMC9573985 36262830

[113] Isaković J, Šerer K, Barišić B, Mitrečić D. Mesenchymal stem cell therapy for neurological disorders: the light or the dark side of the force? Front Bioeng Biotechnol [Internet]. 2023;11. Available from: https://www.frontiersin.org/articles/10.3389/fbioe.2023.1139359/full. 10.3389/fbioe.2023.1139359 PMC10011535 36926687

[114] Petrou P, Kassis I, Ginzberg A, Hallimi M, Karussis D. Effects of mesenchymal stem cell transplantation on cerebrospinal fluid biomarkers in progressive multiple sclerosis. Stem Cells Transl Med [Internet]. 2022;11(1):55–8. Available from: https://academic.oup.com/stcltm/article/11/1/55/6528889. 10.1093/stcltm/szab017 PMC8895488 35641166

[115] Uccelli A, Laroni A, Brundin L, Clanet M, Fernandez O, Nabavi SM, et al. MEsenchymal stem cells for multiple sclerosis (MESEMS): a randomized, double blind, cross-over phase I/II clinical trial with autologous mesenchymal stem cells for the therapy of multiple sclerosis. Trials [Internet]. 2019;20(1):263. Available from: https://trialsjournal.biomedcentral.com/articles/10.1186/s13063-019-3346-z. 10.1186/s13063-019-3346-z PMC6507027 31072380

[116] Okon, I. I., Gbayisomore, T. J., Kankam, S. B., & Jalloh, M. Letter to the editor regarding cerebral blood flow role in delineating treatment effect from true tumor progression in glioblastoma multiforme. World neurosurgery 2024;186:269. 10.1016/j.wneu.2024.03.140. 10.1016/j.wneu.2024.03.140 38849997

